# Malignant catatonia due to clozapine withdrawal: A case report and a brief review of the literature

**DOI:** 10.1192/j.eurpsy.2022.1831

**Published:** 2022-09-01

**Authors:** C. Regueiro Martín-Albo, F. Mayor Sanabria, M. Fernández Fariña, M.E. Expósito Durán, A. García Recio

**Affiliations:** Hospital Clínico San Carlos, Institute Of Psychiatry And Mental Health, Madrid, Spain

**Keywords:** Catatonia, clozapine, withdrawal

## Abstract

**Introduction:**

Withdrawal symptoms are common upon discontinuation of many psychotropic drugs. Catatonia, a neuropsychiatric condition characterized by a number of motor, behavioral, emotional, and autonomic abnormalities, has been described as a withdrawal syndrome in a growing number of case reports, but it is not well recognized. Treatment of catatonia usually includes benzodiazepines and electroconvulsive therapy. Standard consensus states that the use of neuroleptics should be avoided, as they are thought to worsen catatonia.

**Objectives:**

With this case report, we attempt to contribute to the finding in literature that the withdrawal of clozapine may be associated with catatonia, and how reintroduction of clozapine could be indicated for its treatment.

**Methods:**

A clinical case is presented of a 37-year-old female with a history of schizophrenia, presenting with altered mental status and new onset of catatonic signs and symptoms in the setting of a 7-day emetic syndrome. The possibility that vomiting prevented proper absorption of clozapine is postulated, causing the patient to present clinical features compatible with malignant catatonia.

**Results:**

The patient required treatment with benzodiazepines, electroconvulsive therapy and clozapine re-initiation, leading to improvement of catatonic symptoms within a few days.

**Conclusions:**

This case serves as a reminder to consider alternative diagnostic hypotheses in cases of catatonic syndrome unresponsive to standard treatments. When the clinical suspicion of drug withdrawal is high, restarting the discontinued medication, even an antipsychotic agent, may be indicated.

**Disclosure:**

No significant relationships.

